# Predictors of seropositivity for human herpesvirus type 8 in patients with mild cirrhosis

**DOI:** 10.1038/emi.2017.32

**Published:** 2017-06-07

**Authors:** Kuo-Chih Tseng, Ming-Nan Lin, Tang-Yuan Chu, Jen-Pi Tsai, Cheng-Chuan Su

**Affiliations:** 1 School of Medicine, Tzu Chi University, Hualien 970, Taiwan; 2 Department of Internal Medicine, Buddhist Dalin Tzu Chi Hospital, Chiayi County 622, Taiwan; 3 Department of Family Medicine, Buddhist Dalin Tzu Chi Hospital, Chiayi County 622, Taiwan; 4 Institute of Medical Sciences, Tzu Chi University, Hualien 970, Taiwan; 5 Department of Obstetrics and Gynecology, Buddhist Tzu Chi Medical Center, Hualien 970, Taiwan; 6 Institute of Medicine, Chung Shan Medical University, Taichung 402, Taiwan; 7 Departments of Clinical Pathology and Anatomic Pathology, Buddhist Dalin Tzu Chi Hospital, Chiayi County 622, Taiwan

**Keywords:** alcohol, cirrhosis, HBV, HCV, HHV-8

## Abstract

The high seroprevalence of human herpesvirus type 8 (HHV-8) in moderate or severe cirrhotics appears to be associated with male sex, hepatitis B virus (HBV) infection, alcoholism, and disease severity. The status of HHV-8 infection in mild cirrhotics remains unclear. Plasma samples collected from 93 mild cirrhotics and 93 age- and sex-matched healthy controls were analyzed for HHV-8 antibody and HHV-8 DNA. Mild cirrhotics had higher seropositivity for HHV-8 antibodies than healthy controls (*P*=0.0001). Univariate logistic regression analysis revealed that an age ≥55 years (odds ratio (OR) 2.88, *P*=0.02), hepatitis C virus (HCV) infection (OR 3.42, *P*=0.01), and hepatitis activity (OR 4.10, *P*=0.004) were associated with HHV-8 seropositivity in cirrhotics. Stepwise multivariate logistic regression analysis confirmed that age ≥55 years (adjusted OR (aOR) 1.92, *P*=0.04) and hepatitis activity (aOR 3.55, *P*=0.005) were independent factors. The rate of hepatitis activity was higher in HCV-infected than in HBV-infected patients (*P*<0.0001) and in women than in men (*P*=0.0001). Cirrhotics who were seropositive for HHV-8 or HCV or had hepatitis activity were significantly older (*P*=0.02, <0.0001 and <0.0001, respectively). Plasma samples from all participants were negative for HHV-8 DNA. HHV-8 antibody titers in mild cirrhotics also markedly exceeded those in controls (*P*<0.0001), as did those in patients ≥55 years old vs. younger patients (*P*=0.01), those in patients with vs. without HCV infection (*P*=0.0008), and those in patients with vs. without hepatitis activity (*P*=0.0005). Mild cirrhotics had high HHV-8 seroprevalence and HCV infection, and, in particular, old age and hepatitis activity were predictors.

## INTRODUCTION

To date, HHV-8 DNA has been found consistently in all types of Kaposi’s sarcoma (KS).^1^ This neoplasm occasionally develops in human immunodeficiency virus (HIV) non-infected patients with variable immunologic abnormalities.^2, 3^ Immunologic abnormalities have been documented in cirrhotic patients^4, 5^ and are strongly associated with cirrhosis severity.^5, 6^ In a previous study, we found that the seroprevalence of HHV-8 in patients with moderate or severe cirrhosis was significantly greater than that in healthy controls.^7, 8, 9^ It appeared to be associated with cirrhosis severity, sex, and disease etiologies.^7^ However, the prevalence of HHV-8 infection in patients with mild cirrhosis has not been described, nor is it clear whether HHV-8 prevalence is associated with hepatitis activity. This study aimed to assess the prevalence of HHV-8 infection in Child–Pugh class A cirrhotics with or without hepatocellular carcinoma (HCC) and with or without hepatitis activity and to compare it to the prevalence in healthy individuals and in the class B or C cirrhotics reported previously.

## MATERIALS AND METHODS

### Study subjects and sample collection

After obtaining written informed consent from all participants, plasma samples were collected from 93 healthy controls (60 men and 33 women) and 93 Child–Pugh class A cirrhotics with (23 men and nine women) or without (37 men and 24 women) HCC. The age- and sex-matched healthy controls were selected from persons who received routine health examinations during the same period as the cirrhotics were admitted and were proven to be free of cirrhosis and other serious illnesses. All participants were negative for anti-HIV antibodies.

Cirrhosis was diagnosed by biopsy or by clinical diagnostic criteria, including twice-documented ultrasonographic evidence of a coarse and nodular parenchyma, irregular surface, and dull margins with either splenomegaly, ascites, hepatic encephalopathy or varices.^10^ Disease severity was assessed according to the Child–Pugh scoring system.^11^ Diagnosis of HCC was supported by histologic findings or based on unequivocal imaging and laboratory data following the diagnostic criteria published by the European Association for the Study of Liver Disease in 2001.^12^

Cirrhotics with positive tests for serum hepatitis B virus surface antigen (HBsAg) were considered to be HBV-infected; those with positive serum anti-HCV antibodies were considered to be HCV-infected. Alcohol-related cirrhotics were defined as those who had consumed at least 80 g of alcohol daily for at least the previous five years. Hepatitis activity was defined as a plasma alanine aminotransferase (ALT) level ≥1.5-fold higher than the upper limit of the normal range (ULNR).

**Figure 1:**
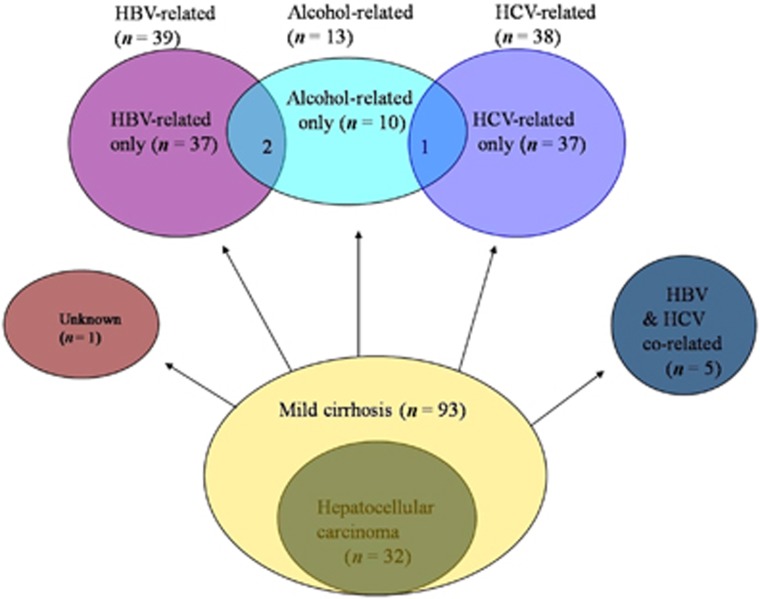
Components of the study group.

Cirrhotics were divided into the following subgroups: HBV-infected (*n*=39; including HBV-infected only (*n*=37) and HBV and alcohol co-related (*n*=2)); HCV-infected (*n*=38; including HCV-infected only (*n*=37) and HCV and alcohol co-related (*n*=1)); HBV and HCV co-infected (*n*=5); alcohol-related (*n*=13; including alcohol-related only (*n*=10), HBV and alcohol co-related (*n*=2), and HCV and alcohol co-related (*n*=1)); and unknown etiology (*n*=1; Figure 1).

The study protocol was approved by the Institutional Review Board of the Buddhist Dalin Tzu Chi Hospital, Taiwan (B09702035).

### Immunofluorescence assay for detection of anti-HHV-8 antibody

A commercially available immunofluorescence assay (IFA) kit (Advanced Biotechnologies, Columbia, MD, USA) was used to detect HHV-8 immunoglobulin G (IgG) antibodies against the lytic antigens in plasma samples according to the manufacturer’s instructions. This assay used HHV-8-infected primary effusion lymphoma cell lines. Human plasma at various dilutions was brought into contact with fixed and infected cells, and was examined with a fluorescence microscope. Samples that displayed fluorescence at a dilution of 1:40 were considered positive. Participants with a positive result by IFA were considered to be HHV-8 positive. Maximum HHV-8 antibody dilutions were determined by an end-point IFA.

### Chemiluminescence immunoassay for HBsAg and anti-HCV and anti-HIV antibodies

Plasma samples were assayed for HBsAg and anti-HCV, anti-HIV-1, and anti-HIV-2 antibodies with the Vitros HBsAg, anti-HCV, and anti-HIV 1+2 reagent packs, respectively, with controls and calibrators (Ortho-Clinical Diagnostics, High Wycombe, England) and the Vitros ECi immunodiagnostic system (Ortho-Clinical Diagnostics, Rochester, NY, USA) according to the manufacturer’s instructions.

### Assay for ALT

Plasma samples were assayed for ALT with the ALT Liquid Reagent with controls and calibrators (Roche Diagnostics, Mannheim, Germany) and the Roche Integra 800 system (Roche Diagnostics, Mannheim, Germany) according to the manufacturer’s instructions.

### DNA extraction and amplification of HHV-8 DNA

HHV-8 DNA was extracted and polymerase chain reaction (PCR)-amplified from the plasma as previously reported.^8^

### Statistical analysis

Differences in the mean values of continuous variables between two groups of patients were analyzed by the *t*-test. A *χ*^2^ or Fisher’s exact test was used to assess the significance of between-group differences in categorical variables. The comparison of anti-HHV-8 titers in plasma between two groups was analyzed by the Mann–Whitney test. Univariate logistic regression was used for analysis of the possible factors predicting the HHV-8 seropositivity in Child–Pugh class A cirrhotics. All variables were entered into stepwise multivariate logistic regression analysis. Statistical significance was set at *P*<0.05. Statistical analyses were performed using SPSS v. 17.0 software for Windows (Chicago, IL, USA).

## RESULTS

### HHV-8 antibody and DNA

The prevalence of HHV-8 antibodies was much greater in mildly cirrhotic patients (46 out of 93) than in healthy controls (21 out of 93) (*P*=0.0001; Table 1). HHV-8 antibody titers in mildly cirrhotic patients also markedly exceeded those in controls (*P*<0.0001; Table 1). The three patients who showed the highest positive dilutions (1:640) were male. Seropositivity was not associated with clinical manifestations of HHV-8 infection, such as KS, primary effusion lymphoma or multicentric Castleman disease.

Cirrhosis patients of both genders had a higher HHV-8 seropositivity (26 out of 60 or 43.3% in male and 20 out of 33 or 60.6% in female patients) than healthy controls (15 out of 60 or 25% in male and six out of 33 or 18.2% in female controls; *P*=0.03 and 0.0004, respectively; *χ*^2^ test). There were no gender differences in HHV-8 seropositivity in either controls or patients, except in the HBV-infected patients for which the rate was significantly greater in female (6 out of 10, 60%) than in male patients (six out of 29, 20.7% *P*=0.02; *χ*^2^ test). Cirrhosis patients who were seropositive for HHV-8 or HCV, or had hepatitis activity, were significantly older (*P*=0.02, <0.0001, and <0.0001, respectively; Table 2). In contrast, HBV-infected cirrhotics were significantly younger (*P*=0.001; Table 2).

We performed HHV-8 DNA PCR analyses on all collected plasma samples. All participants were negative for HHV-8 DNA by this analysis.

### Positivity for HHV-8 antibody by age

Mildly cirrhotic patients 55 years of age or older had higher seropositivity and titers of HHV-8 antibodies than younger patients (*P*=0.01, both *χ*^2^ test and Mann–Whitney test, respectively) (Table 2).

### HHV-8 seropositivity by HCC

HHV-8 seropositivity in cirrhotics without HCC (30 out of 61, 49.2%) was similar to that in HCC patients (16 out of 32, 50% Table 2).

### Positivity for HHV-8 antibody by disease etiology

HHV-8 seropositivity in cirrhotics with HCV infection was significantly greater than in those without (*P*=0.007; *χ*^2^ test; Table 2). In contrast, patients with HBV infection had lower HHV-8 seropositivity than those without HBV infection (*P*=0.008; *χ*^2^ test; Table 2). The seropositive rate in HBV and HCV co-infected patients was 100% (five out of five); the one patient of unknown etiology was also seropositive for HHV-8. The HHV-8 antibody titers in cirrhotics with HCV infection were much greater than in those without HCV infection (*P*=0.0008; Mann–Whitney test; Table 2).

### Hepatitis activity and HHV-8 seropositivity

In 38 HCV-infected patients, 27 had hepatitis activity (71.1%), and 19 of them were seropositive for HHV-8 (70.4%). In contrast, none of the 39 HBV-infected patients had plasma ALT levels ≥1.5-fold higher than ULNR (*P*<0.0001; *χ*^2^ test), and 12 of them were seropositive for HHV-8 (30.8%). Three of the five patients with HBV and HCV co-infection showed hepatitis activity, and none of the 13 alcohol-related patients had plasma ALT levels ≥1.5-fold higher than ULNR. Of 33 female patients, 19 had hepatitis activity (57.6%); in contrast, 11 of 60 male patients had hepatitis activity (18.3%, *P*=0.0001; *χ*^2^ test). In patients without HCC, 17 had hepatitis activity (27.9%, 17 out of 61) and 12 were seropositive for HHV-8 (70.6%, 12 out of 17). In patients with HCC, 13 had hepatitis activity (40.6%, 13 out of 32) and 10 were seropositive for HHV-8 (76.9%, 10 out of 13). Hence, HHV-8 seropositivity was greater in all cirrhotics with hepatitis activity (22 out of 30, 73.3%) compared to those without hepatitis activity (24 out of 63, 38.1% *P*=0.002; *χ*^2^ test; Table 2). The HHV-8 antibody titers in cirrhotics with hepatitis activity were also greater than in those without hepatitis activity (*P*=0.0005; Mann–Whitney test; Table 2).

### Associated factors for HHV-8 seropositivity

Univariate logistic regression analysis revealed that age ≥55 years old (odds ratio (OR) 2.88, *P*=0.02), HCV infection (OR 3.42, *P*=0.01), and hepatitis activity (OR 4.10, *P*=0.004) were predicting factors for HHV-8 seropositivity in Child–Pugh class A cirrhotics (Table 3). Stepwise multivariate logistic regression analysis further confirmed that age ≥55 years old (adjusted odds ratio (aOR) 1.92, *P*=0.04) and hepatitis activity (aOR 3.55, *P*=0.005) were independent predictors (Table 4).

## DISCUSSION

HHV-8 seroprevalence in cirrhotics appeared to be associated with disease severity. In our earlier study concerning patients without hepatitis activity, the seroprevalence of HHV-8 infection in those with Child–Pugh class B cirrhosis (42%) was lower than that in those with class C cirrhosis (47%).^8^ In the present study, among Child–Pugh class A cirrhotics without hepatitis activity, the seropositive rate (38.1%) was even lower.

Our recent study concerning HHV-8 seroprevalence in patients with end-stage renal disease found that one of the two healthy controls with the highest IFA antibody titer (1:160) was positive by enzyme-linked immunosorbent assay.^13^ If the cut-off point of the HHV-8 antibody titer in the present study was set at 1:160, patients with mild cirrhosis still had significantly higher HHV-8 seropositive rate than controls (*P*=0.0003; Table 1).

KS is found mainly in elderly persons, and an age-related slightly increased risk for classic KS has been found among individuals from Asia and Africa.^14, 15^ In the present study, HHV-8-seropositive patients were significantly older than seronegative patients. Patients with HCV infection or hepatitis activity were also significantly older than those without. HHV-8 seropositivity in patients with HCV infection or hepatitis activity was also significantly greater than in those without. In addition, patients aged 55 or older had significantly higher HHV-8 seropositivity than younger patients. Stepwise multivariate logistic regression analysis confirmed that age ≥55 years was an independent risk factor for HHV-8 seropositivity. Thus, old age seems to have an important role in HHV-8 infection in patients with mild cirrhosis, particularly in patients with HCV infection or hepatitis activity.

Mildly cirrhotic patients with HCC in the present study showed similar HHV-8 seropositivity as patients without HCC did. These findings support the hypothesis that HHV-8 seropositivity in cirrhotics is associated with cirrhosis severity, not with HCC.^9^

HHV-8 seropositivity in patients with class A cirrhosis in the present study is greater than in healthy controls and even greater than in patients with class B or C cirrhosis (Table 5). This is mainly attributable to the significantly higher positive rate among HCV-infected patients in this group (63.2%) than in the more severe groups (32%–34%) (Table 5). Abnormal cellular immunities have been found in patients with chronic HCV infection.^16, 17, 18, 19, 20^ Furthermore, inflammatory activity is more important in HCV-infected cirrhotics than in the HBV-infected.^21, 22^ In the present study, many more HCV-infected patients (27 out of 38, 71.1%) showed hepatitis activity than HBV-infected patients (0 out of 39), and HHV-8 seropositivity in patients with hepatitis activity was as high as 73.3%. Hence, the significantly higher seropositivity in patients with mild cirrhosis appears to be associated with the inflammatory properties of HCV.

This significant decrease in HHV-8 seropositivity in HCV-infected patients with more severe cirrhosis (Table 5) indicates that there may be seroreversion to HHV-8 antibody in cirrhotics, particularly in HCV-infected patients. In a study of patients after bone marrow transplantation, half of the 20 recipients who initially had positive results for HHV-8 antibody seroreverted within one year.^23^ The incidence of seroreversion in patients undergoing hemodialysis is 16.4 out of 100 person-years. Patients 50 years of age and younger have an increased probability for seroreversion than those older than 50 years.^24^ However, seroreversion of antibody activity to HHV-8 in patients with cirrhosis has not been described.

It has been reported that all cases with hepatitis activity in asymptomatic HCV carriers occurred during the first 5 years after diagnosis. The presence of the C100-3 antibody and anti-human T cell leukemia virus-I antibody are two independent and significant predictors of hepatitis activity in asymptomatic HCV RNA carriers.^25^ However, no study concerning hepatitis activity or other hepatitis-associated antibodies in HCV-infected mildly cirrhotic patients has been documented. The relationship between HHV-8 antibody and hepatitis activity in mildly cirrhotic patients, particularly in HCV-infected patients, needs to be investigated.

Like HCV infection, patients with chronic HBV infection have been found to have impaired cellular immunities.^26, 27^ In the present study, among HBV-infected low-grade cirrhotics, HHV-8 seropositivity in women was significantly greater than that in men (*P*=0.02). Hence, gender seems to have a role in this subgroup.

In contrast to patients with moderate or severe cirrhosis,^7, 8^ most of the HCV-infected patients with mild cirrhosis in the present study were female (22 out of 38). However, most of the female patients were HCV-infected (22 out of 33). This is compatible with the observation of an increasing HCV prevalence in women, most likely reflecting the increase in hepatitis C over hepatitis B.^22^ Hence, the high HHV-8 seroprevalence in female patients in the present study may be due to the fact that two-thirds of them are HCV-infected. We also found that a significantly larger proportion of female compared to male patients had hepatitis activity, mainly due to a high prevalence of HCV infection. Whether a female sex hormone may contribute to hepatitis activity deserves further exploration.

HHV-8 has two modes of infection—latency and lytic replication. Few viral genes are expressed during latent infection, and the HHV-8 genome is maintained as an episome.^28, 29^ During the lytic cycle, virtually all HHV-8 genes are expressed, resulting in the generation of infectious progeny virions that destroy the host cell.^30^ In the present study, we found anti-lytic phase antibodies at titers as high as 1:640; it is possible that the virus was actively transducing and translating its messages. Many studies have reported that HHV-8 DNA is detected more frequently and at higher copy numbers in the plasma of KS patients compared with controls with asymptomatic HHV-8 infection.^31, 32, 33, 34^ In addition, the method used in this study can only detect 5–10 copies per mL of HHV-8 DNA and may be not sensitive enough to detect low levels of HHV-8 DNA. This may be the reason why none of the plasma specimens were positive for HHV-8 DNA. The question of whether or not patients such as those who participated in this study have HHV-8 DNA at levels less than 5–10 copies per mL will be resolved only if the detection sensitivity can be increased.

HHV-8 is a B-lymphotropic virus. HHV-8 DNA is found principally in circulating B cells in infected subjects.^35, 36^ Hence, HHV-8 DNA may be detected at higher copy numbers in peripheral blood mononuclear cells (PBMCs) than in plasma samples. The second limitation of our study is that no PBMCs from patients were analyzed for HHV-8 DNA. We cannot be sure whether PBMCs from mildly cirrhotic patients contain HHV-8 DNA.

In our study, stepwise multivariate logistic regression analysis also demonstrated that hepatitis activity in mildly cirrhotic patients was an independent predictor for HHV-8 seropositivity. Univariate logistic regression analysis showed that HCV infection was one risk factor able to enhance the prediction of HHV-8 infection. HHV-8 infection appeared to have a role in hepatitis activity in mildly cirrhotic patients. Nevertheless, a third limitation of the present study was that no patients with hepatitis activity received biopsies that proved the presence of HHV-8 DNA in their liver tissues. The role of HHV-8 infection in hepatitis and its influence on the evolution of HCV infection need to be clarified.

In 1972, Triger *et al* demonstrated a highly significant increase in the frequency of high antibody titers (≥1/128) to measles and/or rubella viruses in 15 patients (14 women and one man) with chronic active hepatitis.^37^ Two years later, Laitinen and Vaheri^38^ also reported that seven patients (five women and two men) with chronic active hepatitis had high measles and/or rubella antibody titers without preceding rubella or measles infection. Highly significant increases in high-titer antibodies to HHV-8 in patients with chronic active hepatitis have not been reported to date. Without biopsy in patients with hepatitis activity in the present study, they cannot be proven to have chronic active hepatitis. Therefore, we cannot definitely state whether the increased HHV-8 antibody titers in mildly cirrhotic patients with hepatitis activity are due to atypical viral infections or to atypical immune responses. However, similar to the above two reports, in the present study, most of the mildly cirrhotic patients with hepatitis activity who were seropositive for HHV-8 antibodies were female. Female hormones may have a role in increasing viral antibody titers in these patients. This needs to be further studied.

In conclusion, both the seropositive rates and antibody titers for HHV-8 were increased in patients with mild cirrhosis and were even greater than those in patients with moderate or severe cirrhosis. This indicates that there may be seroreversion to HHV-8 antibodies in moderately or severely cirrhotic patients, particularly in HCV-infected patients. Old age and hepatitis activity are two independent predictors for HHV-8 seropositivity in mildly cirrhotic patients, and HCV infection is a risk factor that can enhance the prediction of HHV-8 infection. The definite relationships between HHV-8 infection and HCV infection, in particular, old age or hepatitis activity in mildly cirrhotic patients, as well as the impact of HHV-8 infection on the disease evolution, remain to be elucidated.

## Figures and Tables

**Table 1:** Mean age and positivity and maximal titers of plasma HHV-8 antibodies in healthy controls and mild cirrhotics

	**Healthy controls (*n*=93)**	**Mild cirrhotics (*n*=93)**	***P*-value**
*Mean age (range)*	56.7 (29–79)	56.7 (27–81)	
			
*IFA+*^a^	22.6% (21/93)	49.5% (46/93)	0.0001^b^
Mean age of IFA+^a^	60.3 (30–79)	59.5 (28–76)	0.80^c^
			
*Anti-HHV-8 titers*			<0.0001^d^
1:40	16	20	
1:80	5	14	
1:160	0	5	
1:320	0	4	
1:640	0	3	
*Rate of high titers^e^*	0% (0/93)	12.9% (12/93)	0.0003^f^

Abbreviations: human herpesvirus type 8, HHV-8; immunofluorescence assay, IFA.

a Positive results of immunofluorescence assay.

b *χ*^2^ test.

c *t*-test.

d Mann–Whitney test.

e HHV-8 antibody titers ≥1:160.

f Fisher’s exact test.

**Table 2:** Comparisons of mean age and maximal titers and positivity of plasma HHV-8 antibodies among various subgroups of patients with Child–Pugh class A cirrhosis

			**IFA maximal titers**								
**Serum dilution**	**Age, y/o** **Mean (range)**	***P*-value**^a^	**Negative**	**1:40**	**1:80**	**1:160**	**1:320**	**1:640**	***P*-value**^b^	**IFA+**^c^	***P*-value**^d^
*Age* ≥*55 y/o*											
Yes	64.8 (55–81)	<0.0001	22	14	9	4	3	3	0.01	33/55 (60%)	0.01
No	44.9 (27–54)		25	6	5	1	1	0		13/38 (34.2%)	

*HCC*											
Yes	59.2 (33–76)	0.15	16	4	6	3	3	0	0.51	16/32 (50%)	0.94
No	55.5 (27–81)		31	16	8	2	1	3		30/61 (49.2%)	

*HBV-related*^e^											
Yes	51.7 (27–70)	0.001	27	8	2	0	2	0	0.07	12/39 (30.8%)	0.008
No	60.1 (33–81)		20	9	11	5	1	3		29/49 (58.2%)	

*HCV-related*^f^											
Yes	62.9 (49–81)	<0.0001	14	6	9	5	1	3	0.0008	24/38 (63.2%)	0.007
No	51.4 (27–70)		33	11	4	0	2	0		17/50 (34%)	

*Alcohol-related*^g^											
Yes	50.6 (33–59)	0.05	8	3	2	0	0	0	0.24	5/13 (38.5%)	0.39
No	57.7 (27–81)		39	17	12	5	4	3		41/80 (51.3%)	

*Hepatitis activity*^h^											
Yes	63.3 (49–76)	<0.0001	8	6	10	3	2	1	0.0005	22/30 (73.3%)	0.002
No	53.5 (27–81)		39	14	4	2	2	2		24/63 (38.1%)	

*IFA+*^c^											
Yes	59.5 (28–76)	0.02	0	20	14	5	4	3		46/46 (100%)	
No	53.9 (27–71)		47							0/47 (0%)	

Abbreviations: hepatitis B virus, HBV; hepatocellular carcinoma, HCC; hepatitis C virus, HCV; human herpesvirus type 8, HHV-8; immunofluorescence assay, IFA; years old, y/o.

a *t*-test.

b Mann–Whitney test.

c Positive results of immunofluorescence assay.

d *χ*^2^ test.

e Including HBV-related only (*n*=37), and HBV- and alcohol-related (*n*=2).

f Including HCV-related only (*n*=37), and HCV- and alcohol-related (*n*=1).

g Including alcohol-related only (*n*=10), HBV- and alcohol-related (*n*=2) and HCV- and alcohol-related (*n*=1).

h Hepatitis activity was defined by plasma alanine aminotransferase level ≥1.5-fold higher than the upper limit of the normal range.

**Table 3:** Predictors for HHV-8 seropositivity in Child–Pugh class A cirrhotics analyzed by univariate logistic regression analysis

	**OR**	**95% CI**	***P*-value**
*Gender*			
Female	1		
Male	0.50	0.21–1.18	0.11

*Age (years old)*			
<55	1		
≥55	2.88	1.22–6.82	0.02

*Etiology*			
HBV	1		
HCV	3.42	1.32–8.91	0.01
Alcohol	1.74	0.44–6.85	0.43

*Hepatitis activity*^a^			
No	1		
Yes	4.10	1.57–10.67	0.004

Abbreviations: confidence interval, CI; hepatitis B virus, HBV; hepatitis C virus, HCV; human herpesvirus type 8, HHV-8; odd ratio, OR.

a Hepatitis activity was defined by plasma alanine aminotransferase level ≥1.5-fold higher than the upper limit of the normal range.

**Table 4:** Predictor for HHV-8 seropositivity in patients with Child–Pugh class A cirrhosis analyzed by stepwise multivariate logistic regression analysis

	**aOR**	**95% CI**	***P*-value**
Age ≥55 years old	1.92	1.03–3.59	0.04
Hepatitis activity^a^	3.55	1.46–8.62	0.005

Abbreviations: adjusted odd ratio, aOR; confidence interval, CI; human herpesvirus type 8, HHV-8.

a Hepatitis activity was defined by plasma alanine aminotransferase level ≥1.5-fold higher than the upper limit of the normal range.

All variables were put into stepwise multivariate logistic regression analysis.

**Table 5:** HHV-8 seropositivities in patients with variant severity of cirrhosis

	**IFA+**^a^	**References**
*Advanced cirrhosis group A (n*=*59)*	25/59 (42%)	7
Child–Pugh class B	9/25 (36%)	
Child–Pugh class C	16/34 (47%)	
HBV-related	11/18 (61%)	
HCV-related	9/28 (32%)	
Alcohol-related	9/18 (50%)	
*Advanced cirrhosis group B (n*=*91)*	41/91 (45%)	8
Child–Pugh class B	16/38 (42%)	
Child–Pugh class C	25/53 (47%)	
HBV-related	14/25 (56%)	
HCV-related	12/35 (34%)	
Alcohol-related	19/37 (51%)	
*Mild cirrhosis (Child–Pugh class A) (n=93)*	46/93 (49.5%)	Present
Without hepatitis activity^b^	24/63 (38.1%)	
With hepatitis activity^b^	22/30 (73.3%)	
HBV-related	12/39 (30.8%)	
HCV-related	24/38 (63.2%)	
Alcohol-related	5/13 (38.5%)	

Abbreviations: hepatitis B virus, HBV; hepatitis C virus, HCV.

a Positive results of immunofluorescence assay.

b Hepatitis activity was defined by plasma alanine aminotransferase level≥1.5-fold higher than the upper limit of the normal range.
